# Manufacturing Differences Affect Human Bone Marrow Stromal Cell Characteristics and Function: Comparison of Production Methods and Products from Multiple Centers

**DOI:** 10.1038/srep46731

**Published:** 2017-04-27

**Authors:** Shutong Liu, Luis F. de Castro, Ping Jin, Sara Civini, Jiaqiang Ren, Jo-Anna Reems, Jose Cancelas, Ramesh Nayak, Georgina Shaw, Timothy O’Brien, David H. McKenna, Myriam Armant, Leslie Silberstein, Adrian P. Gee, Derek J. Hei, Peiman Hematti, Sergei A. Kuznetsov, Pamela G. Robey, David F. Stroncek

**Affiliations:** 1 Cell Processing Section, Department of Transfusion Medicine, Clinical Center; National Institutes of Health, Bethesda, Maryland, USA; 2 Skeletal Biology Section, National Institute of Dental and Craniofacial Research, National Institutes of Health, Bethesda, Maryland, USA; 3 Cell Therapy and Regenerative Medicine, University of Utah, Salt Lake City, Utah, USA; 4 Biomedical Excellence for Safer Transfusion (BEST) group, Darthmouth-Hitchcock Medical Center, One Medical Center Drive, Lebanon, New Hamphire 03756, USA; 5 Hoxworth Blood Center & Department of Pediatrics, The University of Cincinnati, Cincinnati, Ohio, USA; 6 Regenerative Medicine Institute, National Centre for Biomedical Engineering and Science, National University of Ireland Galway, Galway, Ireland; 7 Molecular and Cellular Therapeutics, University of Minnesota, Minneapolis, Minnesota, USA; 8 Production Assistance for Cell Therapies (PACT) group, National Heart, Lung and Blood Program, PACT Coordinating Center, The Emmes Corporation, 401 North Washington Street, Suite 700, Rockville, Maryland 20850, USA; 9 Center for Human Cell Therapy, Programs in Cellular & Molecular Medicine, Boston Children’s Hospital, Boston, Massachusetts, USA; 10 Center for Cell and Gene Therapy, Texas Children’s Hospital, The Methodist Hospital, and Baylor College of Medicine, Houston, Texas, USA; 11 Waisman Biomanufacturing, University of Wisconsin-Madison, Madison, Wisconsin, USA; 12 Department of Medicine, University of Wisconsin-Madison, Madison, Wisconsin, USA; 13 University of Wisconsin Carbone Cancer Center, Madison, Wisconsin, USA

## Abstract

Human bone marrow stromal cells (BMSCs, also known as bone marrow-derived mesenchymal stem cells) are manufactured using many different methods, but little is known about the spectrum of manufacturing methods used and their effects on BMSC characteristics and function. Seven centers using, and one developing, Good Manufacturing Practices (GMP) processes were surveyed as to their production methods. Among the seven centers, all used marrow aspirates as the starting material, but no two centers used the same manufacturing methods. Two to four BMSC lots from each center were compared using global gene expression. Among the twenty-four BMSC lots from the eight centers intra-center transcriptome variability was low and similar among centers. Principal component analysis and unsupervised hierarchical clustering analysis separated all the lots from five centers into five distinct clusters. BMSCs from six of the eight centers were tested for their ability to form bone and support hematopoiesis by *in vivo* transplantation (defining features of BMSCs). Those from all six centers tested formed bone, but the quantity formed was highly variable and BMSCs from only three centers supported hematopoiesis. These results show that differences in manufacturing resulted in variable BMSC characteristics including their ability to form bone and support hematopoiesis.

Bone marrow stromal cells (BMSCs), which are also known as bone marrow-derived mesenchymal stem cells or mesenchymal stromal cells (MSCs), are a heterogeneous population of cells that contain a subset of skeletal stem cells that form cartilage, bone, and support hematopoiesis and formation of marrow adipocytes^1^. BMSCs are being tested in a wide variety of early phase clinical trials. They are most often being tested for the treatment of cardiac, neurological and orthopedic diseases^2^. In addition, BMSCs are being used to treat acute graft versus host disease^3^,^4^,^5^, acute lung injury^6^, ischemic heart disease^7^,^8^ and inflammatory bowel disease^9^,^10^, although long-term outcomes are mostly not available as of yet.

A wide variety of methods can be used to produce BMSCs^2^,^11^ and it is known that some differences in culture conditions affect BMSC quality. Prolonged culture of BMSCs results in cell senescence, but prior to undergoing senescence BMSCs show a number of functional changes including reduced proliferation, reduced secondary colony formation and a reduction in differentiation potential^12^,^13^,^14^,^15^. Furthermore, on a molecular level, changes in BMSCs occur with each passage during culture^16^. It is also known that cells with similar cell surface characteristics from other tissues (loosely termed “MSCs”) have different differentiation potential^17^. For example, when compared with BMSCs, adipose tissue-derived “MSCs” are unable to spontaneously differentiate into bone upon *in vivo* transplantation^18^.

While there are differences in the methods used to produce BMSCs for clinical trials, little is known concerning how much variability exists among academic centers manufacturing BMSCs for early stage clinical trials and how these differences affect product quality. Eight centers including seven manufacturing BMSCs for early phase clinical trials and one developing Good Manufacturing Practice (GMP) methods to produce BMSCs were surveyed concerning the methods that they used to produce BMSCs. We compared 2 to 4 BMSC lots that were manufactured by each center using global gene expression analysis^16^,^19^,^20^,^21^,^22^ and assessed their ability to form bone and support hematopoiesis in an *in vivo* transplant model^23^, both of which are defining features of BMSCs.

## Results

### Comparison of Methods

The methods used by four centers to manufacture BMSCs have been previously reported^24^,^25^,^26^,^27^ and the manufacturing methods of all eight centers are summarized in Table 1. Seven of the eight centers were manufacturing BMSCs using GMP methods while one center (#7), was in the process of developing a GMP method. Seven of the eight centers used bone marrow aspirates from healthy subjects; one center (#7), used marrow from two healthy donors (lots A and C) and one deceased organ donor (lot B). The aspirated marrow was subjected to density gradient separation to remove red blood cells by three centers (#3, #5 and #8), no red blood cell removal was performed by four centers (#1, #2, #4 and #6) and Center #7 tested both methods. Most centers cultured the cells in α-minimum essential medium (α-MEM) and all centers supplemented the media. Fetal bovine serum (FBS) was used as a media supplement by six centers and the final concentration used ranged from 10% to 20%. One (#2) of these six centers used 10% FBS plus fibroblast growth factor (basic) (bFGF). One center (#1) used human platelet lysate and one center (#3) used a serum-free medium supplement (Table 1 and Supplementary Table S1).

All but one center cultured BMSCs in T flasks, multiple layer flasks or both. Center #1 cultured BMSCs in an automated hollow fiber culture system (Quantum Cell Expansion System, Terumo BCT, Lakewood, CO, USA). The duration of the culture and the number of passages used to produce the final BMSC product was, in general, low, but varied among centers (Table 1 and Supplementary Table S1). Five centers used fresh marrow aspirates as the starting material for the samples provided. Center #4 used cryopreserved marrow aspirates; Center #5 provided samples manufactured from fresh aspirates (lots A, B and D) and a sample (lot C) from a cryopreserved intermediate obtained by reseeding lot B; and Center #7 used both fresh marrow aspirates and marrow from cryopreserved deceased organ donor marrow (#7). After the culture was initiated, seven centers passaged all cells through to the final harvest, but Center #8 cryopreserved passage 2 cells and later expanded them to passage 5. Sample data shown in Table 1 and Supplementary Table S1 correspond to information available from each center. Due to donor data protection policies, some centers did not provided information about the gender (Centers #3 and #7) or donor age (Centers #3 and #7), or provided limited information of donor age, based on their protocol inclusion criteria (Centers #5 and #8).

### BMSC Global Gene Expression Analysis

**Figure 1:**
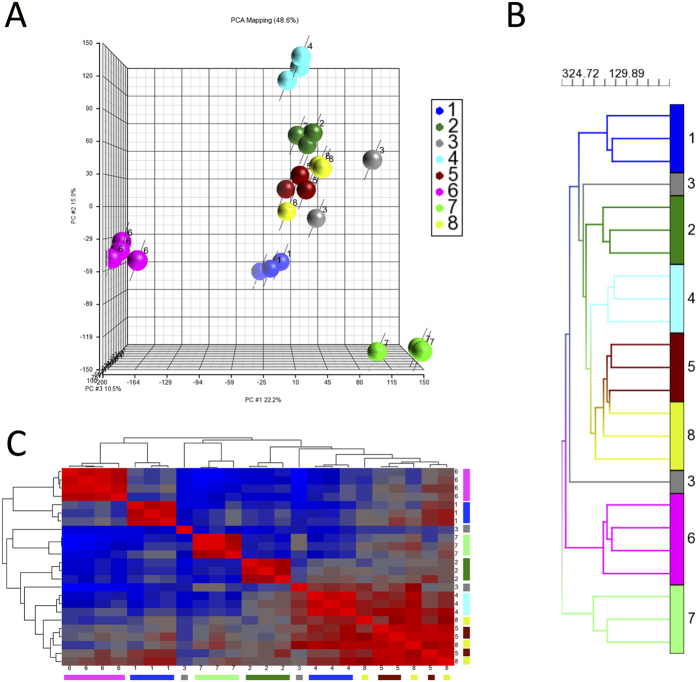
Gene Expression analysis of 24 lots of BMSCs manufactured at 8 different centers. The 24 lots of BMSCs were evaluated by global gene expression analysis and the entire data set was analyzed by PCA (Panel A) and unsupervised hierarchical clustering analysis (panel B). Similarity matrix analysis of all 24 BMSC lots was performed by comparing lots using Pearson correlations and ordering the values using unsupervised hierarchical clustering (Panel C). The samples are color coded according to center.

A total of 26 lots of BMSCs were analyzed by global gene expression analysis. We first assessed 24 lots by principal component analysis (PCA). The 24 lots included 2 to 4 from each center; 3 lots each from six centers (Centers #1, #2, #4, #5, #7 and #8), 2 lots from Center #3 and 4 lots from Center #6. Two of the 26 lots were excluded from this analysis: from Center #6, lot E because it did not meet release criteria^24^, and from Center #5, lot C because it was made from the same donor as lot B. PCA, generally, grouped the 24 lots by center (Fig. 1A).

BMSC lots from Centers #1, #4, #6 and #7 were grouped by center and separate from BMSC lots from the other centers. BMSC lots from Centers #2 and #5 clustered together and BMSCs from Center #8 were grouped among those from Center #5. The 2 lots from Center 3 clustered separately.

Unsupervised hierarchical clustering analysis also grouped the 24 BMSC lots by center except those from Center #3 (Fig. 1B). The BMSCs were separated into 4 major groups. One group was made up of all 3 BMSC lots from Center #7, one group with 4 lots from Center #6, one group with 1 lot from Center #3 and another with the remaining 16 lots. Among the group of 16 lots, those from Centers #1, #2 and #4 were each in a separate subgroup, but BMSCs from Centers #5 and #8 formed a subgroup although within this subgroup the samples segregated by center. The gene expression data from the 24 BMSC lots were subjected to a similarity matrix analysis, which again separated the BMSCs by center except those from Centers #3, #5 and #8 (Fig. 1C).

**Figure 2:**
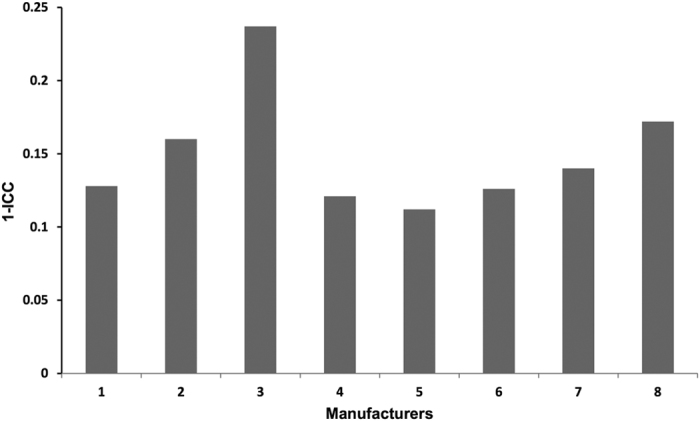
Contributions of intra-center variability on clinical grade BMSCs. The bar chart shows the 1-intraclass correlation coefficient (ICC) values for BMSC lots for each center based on the analysis of the entire transcriptome dataset. The higher the score, the higher the variability between lots.

These results show that inter-center variability in BMSC lots is greater than intra-center variability. To compare intra-center BMSC lot-to-lot variability among the centers, we calculated and compared intra-class correlation coefficients (ICCs) for the eight centers. To assess variability, one BMSC sample from Center #6 was tested three times in the microarray assay and, as expected, the between assay ICC value (0.917) was greater than the intra-center ICC values, which ranged from 0.763 to 0.888, indicating that assay variability was less than intra-center variability. The intra-center ICC values were greatest for centers #1, #4, #5, and #6, and ranged from 0.872 to 0.888, while the ICC values were slightly less for Centers #2, #7 and #8 (0.830 to 0.860) and considerably greater for Center #3 (0.763). The lot-to-lot variability for each center expressed as 1-ICC are shown in Fig. 2. Higher values of 1-ICC indicate greater variability.

**Figure 3:**
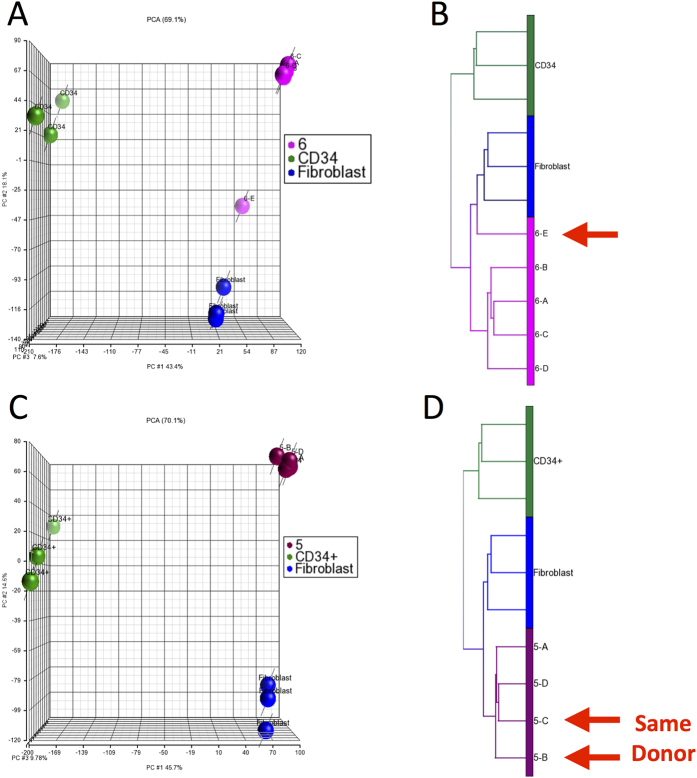
Global gene expression analysis of a lot of BMSCs that failed release criteria and two lots manufactured from the same starting material. Five lots of BMSCs manufactured by Center #6 (magenta) were analyzed by global gene expression analysis. Four of the lots met lot release criteria and are also shown in Fig. 1 and one BMSC lot failed lot release criteria, 6-E (arrow), due to slow growth of the primary culture. The results of PCA are shown in Panel A and unsupervised hierarchical clustering analysis in panel B. A marrow aspirate from one healthy subject was collected and split into multiple aliquots and cryopreserved and on two separate occasions, an aliquot was thawed and used by Center #5 (burgundy) to manufacture BMSCs. These two lots and two others from Center #5 were subjected to global gene expression analysis and PCA is shown in Panel C and unsupervised hierarchical clustering analysis in Panel D. Results of analysis of lots A, B and D from Center 5 (5-A, 5-B and 5-D) are also shown in Fig. 1. The results of analysis of fibroblasts (blue) and CD34^+^ cells (green) from healthy subjects are shown as controls.

We next investigated factors that might contribute to inter- and intra-center variability. These factors included differences in the starting material and in manufacturing. To investigate the effects of the quality of the starting material on the final BMSC product, we analyzed one lot of BMSCs from Center #6 (lot E) which did not meet release criteria because it failed to reach 70 to 80% confluence after 13 days in primary culture^24^; this was likely due to poor quality of the starting material. The BMSCs from this lot were continued in culture using Center #6’s usual manufacturing procedures, but the final product was not released for clinical use. When this BMSC lot was subjected to gene expression and PCA analysis and compared with the 4 previously analyzed lots of BMSCs from Center #6 that met all lot release criteria, the failed lot clustered separately from the other 4 lots (Fig. 3A). Hierarchical clustering analysis yielded similar results (Fig. 3B). These results show that differences in starting material can contribute to BMSC variability.

We also compared one BMSC lot from Center #5, lot C, made from the same cryopreserved marrow aspirate used to make one of the 3 previously analyzed BMSC lots from Center #5, lot B. PCA (Fig. 3C) and unsupervised hierarchical clustering analysis (Fig. 3D) of the 4 BMSC lots from Center 5 showed that all clustered tightly together. These results provide evidence that standardized and controlled manufacturing procedures result in consistent BMSC products.

Specific information concerning donor gender was available for 20 lots and age for 14 of the 20. PCA and unsupervised hierarchical clustering analysis of the 20 lots revealed that they sorted by center rather than age or gender (Supplementary Figure S1A and B). Analysis of the gene expression data from the 14 lots by 3-way ANOVA revealed that inter-center differences made the greatest contribution of transcriptome variations, while age or gender made little contribution (Supplementary Figure S1C). Further comparison of the transcriptomes based on donor gender found that no genes were differentially expressed among males (n = 14) and females (n = 6), nor among donors ≤ 25 years of age (n = 6) and > 25 years (n = 8) (t-tests with a False Discovery Rate adjusted p value < 0.01).

### Evaluation of the Expression of Genes Affecting BMSC Function

**Figure 4:**
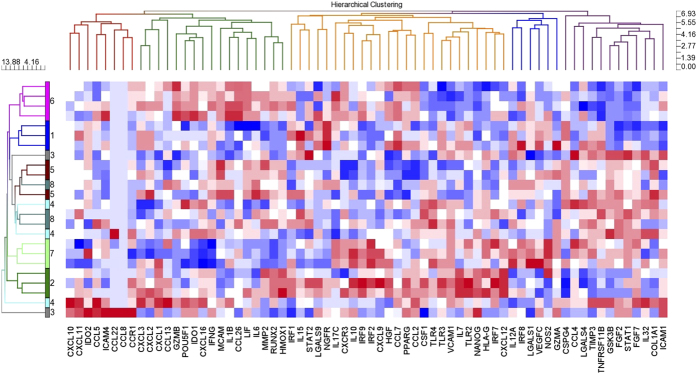
Comparison of the expression of BMSC function genes among the 24 lots of BMSCs. Ninety-three genes reported to be involved with BMSC function or potency were selected from the gene expression data set (Supplementary Table S2) and were analyzed by unsupervised hierarchical clustering.

To determine if inter-center manufacturing differences in BMSCs might affect BMSC function, we compared the expression of 93 genes that have been described as important in BMSC function or as BMSC potency markers (Supplementary Table S2) among the 24 BMSC lots. Unsupervised hierarchical clustering analysis of the 24 BMSC lots using the 93 function and potency genes separated some BMSCs lots by center; those from Centers #1, #2, #6 and #7 (Fig. 4). Lots from Centers #3, #4, #5 and #8 were mixed among lots from other centers or clustered alone. These results suggest the function of BMSCs from Centers #3, #4, #5 and #8 may be similar and that functional differences may exist among BMSCs produced at Centers #1, #2, #6 and #7.

While BMSC genes that are considered to be important in immune modulation, angiogenesis, bone and cartilage formation and progenitor cell function were included in this analysis, there was no segregation of genes by function. Hierarchical clustering analysis separated the genes into 5 nodes, but immune, stem cell and growth factor genes were distributed throughout all of the nodes (Fig. 4).

### *In vivo* Bone Formation and Support of Hematopoiesis

Sufficient numbers of BMSCs were available from 6 of the 8 centers to test for bone formation and support of hematopoiesis in an *in vivo* ectopic bone transplant model. Each BMSC lot was assessed for bone formation and for the areas of marrow covered by both hematopoietic progenitor cells and adipose tissue. BMSC lots from Centers #1, #2, #4, #5, #6 and #8 were thus tested.

**Figure 5:**
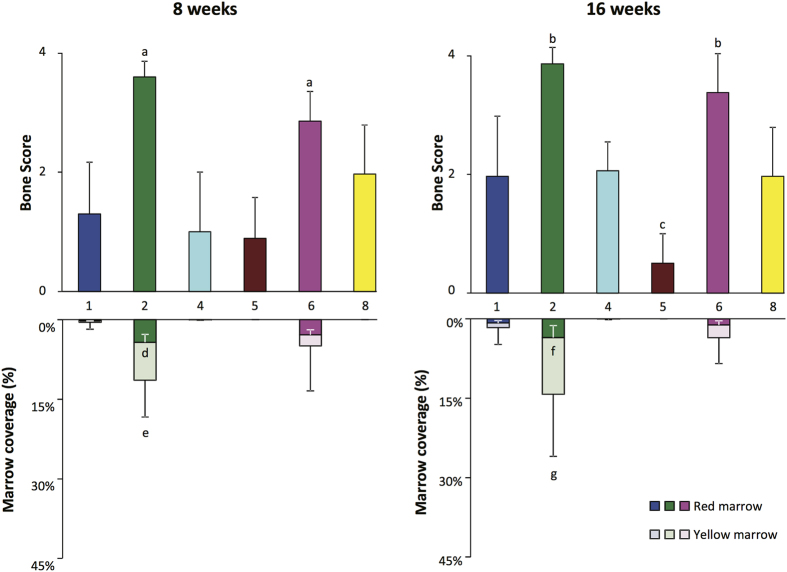
Bone formation, support of hematopoiesis and adipose tissue formation for BMSC lots from each of the 6 centers tested using the ectopic bone transplantation model in immunocompromised mice. The bone formation scores are shown in the upper portion of each panel and the area of marrow coverage by hematopoietic progenitor cells (red marrow) and adipose tissue (yellow marrow) are shown in the lower portion of each panel. The results of analysis 8 weeks after transplantation are shown in the left panel and 16 weeks after transplantation in the right panel. The values shown represent the mean ± SD. “a” is p < 0.01 vs. Centers #1, #4, #5 and #8; “b” is p < 0.01 vs. Centers #1, #4, #5 and #8; “c” is p < 0.05 vs. Centers #1, #4 and #8; “d” is p < 0.05 vs. Center #6; “e” is p < 0.01 vs. Center #1 and p < 0.05 vs. Centers #4 and #5; “f” is p = 0.062 vs. Center #1; “g” is p < 0.01 vs. Center #4, #5 and #8; and p < 0.05 vs. Center #1.

Eight weeks after transplantation, BMSCs from all 6 centers produced some bone, but the results differed significantly among the centers (Fig. 5) (p < 0.0001, ANOVA). The bone scores by transplanted BMSCs from Centers #2 and #6 were greater than those by the other 4 centers (p < 0.01,Tukey’s post-test). After 16 weeks, the bone scores for transplanted BMSCs from all centers increased except for those from Center #5 (Fig. 5 and Supplementary Figure S2). The 16 week bone scores were also variable among the centers (p < 0.0001, ANOVA) and the scores for Centers #2 and #6 remained greater than those of the other 4 centers (p < 0.01, Tukey’s post-test) while those of Center #5 fell below those of Centers #1, #4 and #8 (p < 0.05, Tukey’s post-test).

**Figure 6:**
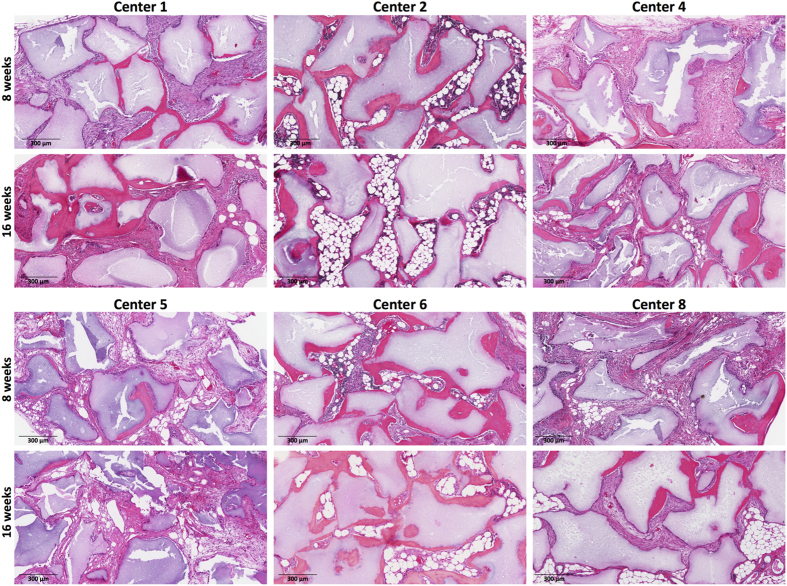
Representative transplant hematoxylin and eosin staining of transplants from each of 6 centers whose BMSCs were tested in the ectopic bone transplantation model on immunocompromised mice. For each center, sections obtained after 8 weeks are shown in the upper panels and for 16 weeks in the lower panels.

Although BMSCs from all centers formed bone, only those from 3 centers, (Centers #1, #2 and #6), supported a significant amount of hematopoiesis and adipose tissue formation (Fig. 4). Analysis at 8 weeks revealed that the area of marrow coverage by transplanted BMSCs from Center #2 was greater than that of Centers #1, #4 and #5. At 16 weeks, the coverage of marrow by hematopoietic progenitor cells and adipose tissue was similar to the week 8 results (Fig. 5). The area of marrow coverage in the transplants of BMSCs from Center #2 was greater than those of Centers #1, #2, #5 and #8. The results of analysis of individual BMSC lots at 8 and 16 weeks are shown in Supplementary Figure S3. The bone score and marrow area score results for each of the duplicate or triplicate transplants of each BMSC lot are show in Supplementary Figure S4). Representative results of transplantation of BMSC lots from centers with high and low bone formation and areas of coverage by hematopoietic cells and adipose tissue are shown in Fig. 6.

**Figure 7:**
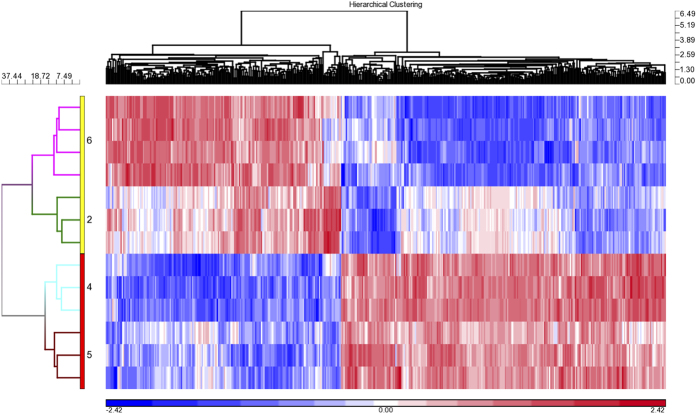
Unsupervised hierarchical clustering analysis of genes differentially expressed among BMSC lots from two centers with high bone formation and support of hematopoiesis scores and two centers with low bone formation scores and no support of hematopoiesis. A total of 455 genes were differentially expressed among BMSCs for the two centers with high scores, Centers #2 and #6, and two centers with low scores, Centers #4 and #5 (p < 0.05 and False Discovery Rate < 0.05).

In order to determine which factors may be responsible for better bone formation and support of hematopoiesis, we compared the transcriptomes for BMSC lots from the two centers with the highest bone formation and support of hematopoiesis scores, Centers #2 and #6, to the two centers with the lowest scores, Centers #4 and #5. A total of 455 genes were differentially expressed between the two groups (p < 0.05 and FDR < 0.05). Unsupervised Hierarchical Clustering analysis using these 455 genes separated the BMSCs lots into two major clusters, one with BMSCs from Centers #2 and #6 and another with those from Centers #4 and #5 (Fig. 7). Within the two major clusters, the BMSCs were separated by center.

Ingenuity Pathway Analysis showed that 65 pathways were enriched with the 264 genes up-regulated in BMSC lots that had reduced bone formation and support of hematopoiesis scores including several immune-related pathways: CXCR4 Signaling, Chemokine Signaling, Integrin Signaling, Jak/Stat signaling and CCR3 Signaling in Eosinophils (p < 0.01) (Supplementary Table S3). BMSC lots that had reduced bone formation and support of hematopoiesis scores were also enriched with genes for ephrin receptor signaling, ephrin B signaling, and RhoA signaling pathways. Ephrin-B1, Ephrin-B2 and RhoA are involved with osteogenic and chrondrogenic differentiation of BMSCs^28^,^29^. Only one pathway was over represented (Sulfite Oxidation IV, p < 0.01) among the 191 genes that were up-regulated in BMSC lots with better bone formation and support of hematopoiesis scores.

## Discussion

We compared BMSC manufacturing methods among 8 academic cell therapy centers and compared human BMSC lots manufactured at each center. We found that none of the 8 centers used exactly the same manufacturing methods, but there were many commonalities. All centers used marrow as a starting material and maintained cells in culture for a relatively short period of time. Most centers cultured the cells in T flasks and/or multilayer flasks. The centers were approximately equally split as to whether they directly cultured marrow aspirates or they cultured mononuclear cells obtained from density gradient separated marrow. Most cultured the cells in α-MEM but there was considerable variation in the type and concentration of media supplements. FBS, human platelet lysate, bFGF and defined media were used. Several different concentrations of FBS and platelet lysate were also used.

Mendicino *et al*.^2^ and Sharmar *et al*.^11^ have recently summarized the methods used to manufacture BMSCs, and the differences in manufacturing methods among centers in the current study were relatively minor compared to those reported in the two reviews. All 8 centers in this study are in some way affiliated with an academic center, which may account for some of the similarities in manufacturing methods. It is likely that greater differences in manufacturing methods would have been found if the study had included centers manufacturing BMSCs commercially.

The results of transcriptome analysis of BMSC lots from the 8 centers revealed that intra-center variability was low. Interestingly, BMSC intra-center manufacturing variability was low but greater than that of the intra-center manufacturing variability of mature dendritic cells (DCs)^20^. We found that 1-intraclass correlation coefficient (ICC) for 5 of the 8 centers ranged from 0.112 to 0.128 which was slightly greater than what was previously found for mature DCs manufactured at a single center, where 1-ICC was equal to 0.075^20^. The fact that BMSC lots manufactured at the same center and from the same donor were grouped together by both PCA and unsupervised hierarchical clustering analysis together with two other lots manufactured by the same center provided further evidence that intra-center variability was relatively low.

Despite the commonalities in manufacturing considerable inter-center variability was noted. Inter-center variability in BMSC manufacturing was much greater than intra-center variability as demonstrated by the separation of BMSC lots by center using PCA and hierarchical clustering analysis. The inter-center variability documented by global gene expression analysis likely reflects variability in function. We found that there was considerable variability among the BMSCs from different centers in their ability to form bone and support hematopoiesis in an *in vivo* transplant model. Since Kuznetsov *et al*. have previously found that human BMSCs that support bone formation and hematopoiesis in a similar transplant model also support bone formation in large animal models (canines)^30^, the results of this study suggest that if the BMSCs from the centers studied were used to repair bone in a clinical trial, there would be considerable differences in the clinical outcomes. Other functional differences may exist among the BMSCs from the 8 centers. We found that when BMSC lots from centers with high and low bone formation and support of hematopoiesis scores were compared, the genes whose expression differed among the two groups were over represented in a number of immune related Ingenuity pathways.

Information concerning age and gender was available for some but not all of the marrow donors. PCA, unsupervised hierarchical clustering analysis and ANOVA analysis of the transcriptome of these samples suggest that differences among centers had a greater influence on BMSC characteristics than age or gender. However, since the donors were of similar age and the number of samples studied was relatively small, we cannot exclude the possibility that age and gender effect BMSC characteristics.

It is not certain which manufacturing factors contributed to the variability among the BMSC lots. Factors that may have contributed to the variability include differences in marrow preparation, media supplements, culture conditions, culture vessels and duration of culture. However, a study which included a much larger number of centers and focused on fewer variables would be required to determine the relative importance of these manufacturing differences on the function of BMSCs. Until the variables that contribute most to BMSC function are identified, clinical trials using BMSCs manufactured at multiple sites should use the same well defined protocol and reagents to manufacture the BMSCs.

In conclusion, centers using GMP manufacturing methods and practices produced BMSC lots with consistent characteristics, but differences in manufacturing methods among centers resulted in considerable differences in BMSC gene expression signatures and function as assessed by bone formation and support of hematopoiesis in an *in vivo* transplant model. It could not be determined which differences in manufacturing contributed to the functional differences. The results suggest that when BMSCs manufacturing changes are necessary, BMSCs manufactured using the original and changed methods should be thoroughly investigated to ensure that the changes do not affect the BMSC functions of clinical interest.

## Methods

### Study Design

All eight of the laboratories were manufacturing BMSCs for use in clinical trials or were validating a method for GMP manufacturing except for Center #7, which was developing a GMP method for the manufacture of BMSCs. The cells were manufactured at each center and were cryopreserved. Each center manufactured BMSCs from unique marrow samples. Cyropreserved samples from 2 to 4 BMSC lots from each center were shipped to one center for gene expression analysis and evaluation by an *in vivo* transplant model (NIH, Bethesda, Maryland, USA). All lots used were evaluated by the manufacturing center and meet their lot release criteria unless otherwise noted. The lot release criteria used varied among centers but included analysis by flow cytometry and analysis of viability. These studies were approved by an Institutional Review Board (IRB) of NHLBI, NIH. The CD34^+^ cell samples used as controls for some studies were isolated using monoclonal antibodies conjugated to magnetic particles (CliniMACS Plus, Miltenyi Biotec GmbH, Bergisch Gladbach, Germany) from G-CSF-mobilized peripheral blood concentrates collected by apheresis from healthy subjects in accordance with American Association Blood Bank standards using a protocol approved by the NHLBI IRB. Fibroblasts (Human Neonatal Foreskin Fibroblasts, from GlobalStem, Rockville, MD, USA), CRL2352 and CRL2429 (Human fibroblast, from ATCC, Manassas, VA, USA)] were also used as controls for some gene expression studies. Informed consent was obtained from all human subjects. All animals were cared for according to the policies and principles established by the Animal Welfare Act and the NIH Guide for the Care and Use of Laboratory Animals. Operations were performed in accordance to specifications of an approved NIH small-animal protocol (NIDCR ASP #13-694).

### Total RNA Purification, Amplification, Hybridization and Microarray Slide Processing

Total RNA from BMSCs and control samples were purified using miRNA Easy Kit (Qiagen, Germantown, MD, USA). The RNA concentration was measured using a Nano Drop ND-1000 Spectrophotometer (Nano Drop Technologies, Wilmington, DE, USA) and RNA quality was assessed with an Agilent 2100 Bioanalyzer (Agilent Technologies, Santa Clara, CA, USA). RNA was amplified using an Agilent LowInput QuickAmp Labeling Kit and subsequently hybridized to Universal Human Reference RNA (Stratagene, Santa Clara, CA, USA) with Agilent Chip Whole Human genome, 4 × 44 k slides according to the provided protocol. The slides were incubated for at least 17 hours at 50 °C.

### Microarray Data Analysis

Raw images were obtained by scanning the microarray slides with an Agilent Scan G2505B using Agilent Scan Control software (version 9.5). The images were extracted using the Feature Extraction Software (Agilent Technologies). Partek Genomic Suite 6.4 (Partek Inc., St. Louis, MO, USA) was used for data visualization, identification of differentially expressed transcripts (p-value ≤ 0.05) and hierarchical cluster analysis. The florescence intensity data was transformed to log2 ratios of each sample versus the universal human RNA reference (Stratagene, Santa Clara, CA, USA). Student’s t-tests were used to identify the differentially expressed genes (both p value and False Discovery Rate (FDR) ≤ 0.05). The Ingenuity Pathway Analysis tool (Ingenuity System Inc., Redwood City, CA, USA) was used for analysis of functional pathways. The microarray data has been deposited in GEO (GSE92640).

### *In vivo* Bone Formation

The *in vivo* bone formation assay was performed in an immunocompromised mouse transplant model as previously described^31^. BMSCs were thawed, washed with culture medium used at the NIH center (αMEM, 10 ug/ml Gentamycin, 20% fetal bovine serum). Cells were not further expanded due to the fact that different centers used different growth medium, and it was intended to use the same passage number for transplantation as was used for the microarray analysis. The viability and number of thawed cells were measured using acridine orange (AO) and propidium iodide (PI) staining (Cellometer ViaStain AOPI, Nexcelcom, Lawrence MA) and a Cellometer Auto 2000 system (Nexcelom), and the cells were directly transplanted. 40 mg aliquots of Attrax ceramic (particle size: 0.5–1 mm, NuVasive, San Diego CA) were sterilized in an oven, washed with PBS and placed into 1.8 mL polypropylene cryotubes. 2 × 10^6^ viable BMSCs in 1 mL of growth media were pipetted into the tubes and incubated for 90 min at 37 °C on a slowly rotating platform. The tubes were then centrifuged at 200 g for 60 sec, and the supernatant discarded. The BMSC transplant constructs were maintained at 4 °C until transplantation. Eight week old female immunocompromised SHC mice (Charles River, Wilmington, MA) were used as recipients for the transplants.

Under aseptic conditions, mice were anaesthetized with 2–5% isoflurane and disinfected with betadine and ethanol wipes. Using sterile instruments, a 2 cm cutaneous incision was performed along the sagittal plane on the back of the animals at the level of L1 to L2 vertebrae. Four independent bilateral subdermal pockets were created using round-tipped scissors, and different human BMSCs-ceramic constructs were inserted into each of these pockets. The incision was then closed with two 7 mm autoclips (CellPoint Scientific, Gaithersburg MD). Every mouse carried one control transplant loaded with an aliquot of the same BMSC sample, known from previous experiments to form a complete ectopic ossicle, to monitor for mouse to mouse transplant development variability (data not show) and three transplants from different centers. After 8 or 16 weeks, mice were euthanized by CO_2_ inhalation and the transplants were harvested, fixed with 4% neutral buffered formaldehyde overnight at 4 °C, decalcified with EDTA 250 mM in PBS at 4 °C for approximately 4 weeks and embedded in paraffin. Five μm sections were obtained and stained with Hematoxylin and Eosin. Slides were digitalized using an Aperio Scanscope CS2 system (Leica, Nussloch, Germany). Due to insufficient numbers of cells, Centers #3 and #7 were excluded from the ectopic transplantation assay, and Centers #4 and #5 were only partially assessed. For the remaining centers, three samples were assessed in triplicates at both time points (Supplementary Table S4).

### Bone and Marrow Tissue Analysis

Hematoxylin and Eosin stained slides were analyzed by three independent observers in a blinded fashion to estimate the amount of bone and marrow, marrow adiposity and hematopoiesis. Bone was scored as previously described^32^. Relative marrow content of the transplants was quantified in low magnification images (equivalent to 10x fields) by manually tracing the areas covered by marrow in relation to the total surface of the transplant using Adobe Photoshop CS6 (Supplementary Figure S5). The area of marrow adipocytes and hematopoietic cells was assessed with Marrow Adiposity and Hematopoiesis scoring systems. Marrow Adiposity was determined by a semi-quantitative score (Supplementary Table S5) in higher magnification micrographs (equivalent to 50x fields), covering all the marrow areas present in the transplants and corrected by the marrow area scored in each image relative to the total marrow area of each transplant. An adipose score of 4 indicates that the entire marrow is occupied by adipose tissue and a score of 0 indicates that all the marrow is occupied by hematopoietic cells. The Marrow Adiposity and Hematopoiesis Score system was validated by manually tracing the relative areas of adipose or hematopoietic tissue within the marrow areas in 15 micrographs with different scores using Adobe Photoshop CS6 (Supplementary Figure S6). Marrow Adiposity and Hematopoiesis scores were also transformed into relative areas of coverage by adipocytes (yellow marrow), and hematopoietic cells (red marrow), in order to produce Fig. 5.

### Bone and Marrow Tissue Statistical Analysis

Statistical analysis was performed using GraphPad Prism 6. To assess the difference among groups for bone scores and marrow and adiposity quantification, data normality was determined by Shapiro-Wilk normality test. For normal data sets an ordinary one-way ANOVA was performed, followed by multiple comparisons using Turkey’s test. For non-parametric data sets, the Kruskal-Wallis test was performed, followed by multiple comparisons using Dunn’s tests. To determine the difference in bone and marrow formation and marrow adiposity between the 8 and 16 week time points, samples were subjected to a paired t-test. P-values less than 0.05 were considered significant. Data are shown as the average ± Standard Deviation (SD). The intra-center correlation coefficient was calculated as previously described^20^.

## Additional Information

**How to cite this article:** Liu, S. *et al*. Manufacturing Differences Affect Human Bone Marrow Stromal Cell Characteristics and Function: Comparison of Production Methods and Products from Multiple Centers. *Sci. Rep.* **7**, 46731; doi: 10.1038/srep46731 (2017).

**Publisher's note:** Springer Nature remains neutral with regard to jurisdictional claims in published maps and institutional affiliations.

## Supplementary Material

Supplementary Tables and Figure Legends

## Figures and Tables

**Table 1:** Comparison of BMSC manufacturing methods used by the 8 Centers.

Center	Donor Type	Marrow Source	Density Gradient Separation	Culture Media	Vessels	Number of Passages
1	Healthy Subjects	Marrow Aspirate	No	DMEM* 5% Human Platelet Lysate 2 mM GlutaMax 10 mM N-acetyl cysteine 2 IU/mL heparin	Quantum Bioreactor	2
2	Healthy Subjects	Marrow Aspirate	No	Alpha MEM^#^ 10% FBS^+^ 5 ng/mL bFGF GlutaMax	T-Flasks	3
3	Healthy Subjects	Marrow Aspirate	Yes	IMDM* 10% “hBM MSC” Supplement^	T-Flasks	4
4	Healthy Subjects	Cryopreserved Marrow Aspirate	No	Alpha MEM 10% FBS^+^ Glutamax	T-Flasks	3
5	Healthy Subjects	Marrow Aspirate	Yes	Alpha MEM 16.5% FBS^+^ 1xGlutaMax	T-Flasks and Multiple Layer Flasks	2
6	Healthy Subjects	Marrow Aspirate	No	Alpha MEM with UltraGlutamine 20% FBS^+^	T-Flasks and Multiple Layer Flasks	4
7	Healthy Subjects and Organ Donors	Fresh or Cryopreserved Marrow Aspirate	Yes and No	Alpha MEM 10% FBS^+^ GlutaMax	T-Flasks	2 to 3
8	Healthy Subjects	Marrow Aspirate	Yes	Alpha MEM 10% FBS^+^ GlutaMax	T-Flasks and Multiple Layer Flasks	5

*Dulbecco’s Modified Eagle Medium.

^#^Minimum Essential Medium.

*Iscove’s Modified Dulbecco’s Media.

^Stem Cell Technologies.

^+^FBS was not heat-inactivated.
