# Supplementary material for: Manufacturing Differences Affect Human Bone Marrow Stromal Cell Characteristics and Function: Comparison of Production Methods and Products from Multiple Centers
Source: Sci Rep. 2017 Apr 27;7:46731. doi: 10.1038/srep46731 (PMC5406832; doi:10.1038/srep46731)
Supplement: Supplementary Tables and Figure Legends [file srep46731-s1.pdf]

Manufacturing Differences Affect Human Bone Marrow Stromal Cell Characteristics and Function: Comparison of Production Methods and Products from Multiple Centers

Shutong Liu[1*], Luis F. de Castro[2*], Ping Jin[1], Sara Civini[1], Jiaqiang Ren[1], Jo-Anna Reems[3, 12], Jose Cancelas[4, 12], Ramesh Nayak[4], Georgina Shaw[5], Timothy O'Brien[5, 12], David H. McKenna Jr[6, 12], Myriam Armant[7], Leslie Silberstein[7], Adrian P. Gee[8], Derek J. Hei[9], Peiman Hematti[10,11], Sergei A. Kuznetsov[2], Pamela G. Robey[2], David F. Stroncek[1, 12]

[1]Cell Processing Section, Department of Transfusion Medicine, Clinical Center; National Institutes of Health, Bethesda, Maryland USA; [2]Skeletal Biology Section, National Institute of Dental and Craniofacial Research, National Institutes of Health, Bethesda, Maryland. [3]Cell Therapy and Regenerative Medicine, University of Utah, Salt Lake City, Utah, USA; [4]Hoxworth Blood Center & Department of Pediatrics, The University of Cincinnati, Cincinnati, Ohio, USA; [5]Regenerative Medicine Institute, National Centre for Biomedical Engineering and Science, National University of Ireland Galway, Galway, Ireland; [6]Molecular and Cellular Therapeutics, University of Minnesota, Minneapolis, Minnesota, USA; [7]Center for Human Cell Therapy, Programs in Cellular & Molecular Medicine, Boston Children's Hospital, Boston, Massachusetts, USA; [8]Center for Cell and Gene Therapy, Texas Children's Hospital, The Methodist Hospital, and Baylor College of Medicine, Houston, Texas, USA; [9]Waisman Biomanufacturing, University of Wisconsin-Madison, Madison, Wisconsin, USA; [10]Department of Medicine, University of Wisconsin-Madison, Madison, Wisconsin, USA and [11]University of Wisconsin Carbone Cancer Center, Madison, Wisconsin, USA and [12]Biomedical Excellence for Safer Transfusion (BEST) group and the Production Assistance for Cell Therapies (PACT) group.

*Co-first authors making equal contributions

Address all correspondence to:

David F. Stroncek, MD

Cell Processing Section

Department of Transfusion Medicine

Clinical Center, NIH

10 Center Drive-MSC-1184

Building 10, Room 3C720

Bethesda, MD 20892-1184

Phone:301-402-3314

dstroncek@cc.nih.gov

Running title: Manufacturing Differences Effect BMSC Function

Supplementary materials

Supplementary Table S1. Detailed sample information.

Center	Lot	Gender	Age	BM aspirate	Dens gradient	Culture media	Vessel	Days cultured	Passages	Cell number in product	Observations
#1	A	Male	32		No	DMEM, 2mM GlutaMax<br>5% Platelet Lysate<br>10mM N-acetyl cysteine<br>2IU/mL heparin	Quantum Bioreactor	6	2	5.5 x 10^8 cells	
	B		19					7		6.25 x 10^8 cells	
	C	Female	27							3.65 x 10^8 cells	
#2	A	Female	25	10 mL	No	Alpha MEM<br>10% FBS<br>5 ng/mL bFGF<br>GlutaMax	T-Flasks		3		Caucasian donors
	B		26								
	C	Male	47								
#3	A				Yes	IMDM<br>10% “hBM MSC” Supplement#	T-Flasks	16-20	4		
	B							16-20	10		
#4	A	Male	20	30 mL – Cryopreserved	No	Alpha MEM<br>10% FBS<br>Glutamax	T-Flasks	26	3	7.20 x 10^7 cells	CFE= 440 CFU per mL of aspirate
	B	Female	19					24		3.69 x 10^8 cells	CFE= 369 CFU per mL of aspirate
	C	Male	26					28		1.19 x 10^8 cells	CFE= 440 CFU per mL of aspirate
#5	A	Male	18-45		Yes	Alpha MEM<br>16.5% FBS<br>GlutaMax	T-Flasks Multiple Layer Flasks	21	2	9.31 X10^8 cells	Cell product is CD14-, CD19-, CD34-, CD45-, CD73+, CD90+, CD105+, HLA-DR+, 7AAD+
	B*							20		2.89 X10^9 cells	
	C*							30	2+1 ^{X}	2.33 X10^9 cells	
	D	Female						28	2	3.29 X10^9 cells	
#6	A	Male	26.2	3.97 X10^8 cells	No	Alpha MEM with UltraGlutamine<br>20% FBS	T-Flasks Multiple Layer Flasks	26	4	3.228X10^9 cells	PDT= 27.68 hrs.
	B		27.6	3.76X10^8 cells						1.606 X10^9 cells	PDT= 36.06 hrs.
	C		32.1	2.21X10^8 cells						2.179X10^9 cells	PDT= 31.85 hrs.
	D		23.7	7.25X10^8 cells				25		2.225X10^9 cells	PDT= 31.6 hrs.
	E$	Female	25.7	3.51X10^8 cells				26		2.17X10^8 cells	PDT=261.00 hrs.
#7	A				Yes	Alpha MEM<br>10% FBS<br>GlutaMax	T-Flasks	20	2	7 x 10^7 cells	
	B							27	3	7 x 10^7 cells	Cryopreserved marrow, cadaveric donor
	C				No			24	3	4.5 x 10^7 cells	
#8	A	Male	18-45	1.40 X10^8 cells	Yes	Alpha MEM<br>10% FBS<br>GlutaMax	T-Flasks Multiple Layer Flasks	21+20^X	2+3^X	3.70 x 10^7 cells	Cryopreserved intermediate
	B	Female	22	4.10 X10^8 cells				19+20^X		3.90 x 10^7 cells	
	C	Male	18-45	3.00 X10^8 cells				14+18^X		1.20 x 10^9 cells	

*Samples center #5 lot B and C were obtained from the same donor; lot C underwent an intermediate cryopreservation process. [$]Sample 6E did not meet release criteria. [#]Stem Cell Technologies. [X]Second number refers to passage number after cryopreserved intermediate. DMEM is Dulbecco's Modified Eagle Medium. MEM is Minimal Essential Medium. IMDM is Iscove's Modified Dulbecco's Media. CFE is colony forming efficiency, PTD is population doubling time. Blank cells indicate that information was not provided by the center

Supplementary Table S2. Biomarkers that have been used to assess BMSC potency or have been found to be important in BMSC function.

Gene	Reference
ADAMTS9	1
Angiogenin	2
Angiopoietin-1 (Ang-1)	2
Angiopoietin-2 (Ang-2)	2
CD271	3
Colony stimulating factor-1 (CSF1) (MCSF)	1
CCL2	4
CCL4	4
CCL5	4
CCL7	4
CCL8	4
CCL13	4
CCL22	4
CCL26	4
CCR1	5
CXCL1	4
CXCL3	4
CXCL7 Neutrophil activating protein 2 (NAP-2)	2
CXCL9	4
CXCL10	4
CXCL11	4
CXCL12	1
CXCL16	4
CXC chemokine receptor-4 (CXCR4)	6
Dendrocyte expression 7 transmembrane protein (TM7SF4)	1
EYA transcriptional coactivator and phosphatase 4 (EYA4)	1
Fibroblast Growth Factor-2 (FGF-2)	2
Fibroblast Growth Factor (basic) (bFGF)	7
Galectin-1 (LGALS1)	8
Galectin-3	9
Galectin-9 (LGALS9)	10
Glycogen synthase kinase-3β (GSK3B)	11
Granzyme A (GZMA)	4
Granzyme B (GZMB)	4
Heme oxygenase 1 (HMXO1)	12
Hepatocyte growth factor (HGF)	2, 13
Human leukocyte antigen G (HLA-G)	4, 14
ICAM-1	1
ICAM-4	4
Indolamine 2,3-dioxygenase-1 (IDO-1)	4, 15
IDO-2	4
Inducible nitric oxide synthase (iNOS)	11
Interferon-γ (IFNG)	4
Interferon regulatory factor 1 (IRF1)	4
IRF2	4
IRF7	4
IRF8	4
IRF9	4
Insulin-like-growth factor 1 (IGF1)	2

IL-1β	4
IL-5	4
IL-6	1, 2
IL-7	4
IL-8	1, 2
IL-10	16
IL-12	4
IL-15	4
IL-17	4
IL-18	4
IL-32	4
Interleukin-1 receptor-associated kinase 3 (IRAK3)	1
Keratinocyte growth factor (KGF)	17
Kruppel-like factor-8 (KLF8)	1
Leukemia inhibitory factor (LIF)	18
Melanocyte cell adhesion molecule (CD146)	19
Macrophage Inflammatory Protein-1alpha (MIP-1α)	2
Macrophage Inflammatory Protein-1alpha (MIP-1β)	2
Monocyte Chemoattractant Protein-1 (MCP-1)	2
Monocyte induced by Interferon-gamma (MIG)	2
Nanog	20, 20, 21
Neuron-glial antigen 2 (NG2)	19
Oct4	20, 21
Osteoprotegerin	22
Platelet derived growth factor receptor (PDGFR)	23
Placental Growth Factor (PLGF)	2
Prostaglandin E2 (PGE2)	24
Rho GTPase activating protein 29 (ARHGAP29)	1
RUNX2	1
Secreted phosphoprotein (SPP1)	1
Serum amyloid A1 (SAA1)	1
Sodium channel, voltage-gated, type IX, alpha subunit (SCN9A)	1
STAT-1	4
STAT-2	4
TEK	1
Tissue inhibitor of matrix metalloproteinase-1 (TMP-1)	2
Tissue inhibitor of matrix metalloproteinase-2 (TMP-2)	2
Tissue inhibitor of matrix metalloproteinase-3 (TMP-3)	25
Toll-like receptor 2 (TLR2)	26, 27
Toll-like receptor 3 (TLR3)	26, 27
Toll-like receptor 4 (TLR4)	26, 27
Transforming growth factor-β (TGFB)	1, 2, 11
Tumor necrosis factor superfamily, member 10 (TNFSF-10)	1
Tumor necrosis factor superfamily, member 11b (TNFSF-11b)	1
Tumor necrosis factor-stimulated gene-6 (TSG-6)	28
Type I collagen (COL1A1)	22
Vascular endothelial growth factor (VEGF)	29, 30
VEGF	1
WISP1	1

Supplementary Table S3. Ingenuity Pathway Analysis of 265 genes that were up-regulated in BMSC lots from 2 centers with the lowest bone formation and support of hematopoiesis scores compared with the 2 centers with highest scores.

**Pathways enriched with genes over-represented in BMSC lots with low scores***	
tRNA Charging	Molecular Mechanisms of Cancer
ERK5 Signaling	Superpathway of Inositol Phosphate Compounds
Ephrin Receptor Signaling	IGF-1 Signaling
Thrombin Signaling	Myc Mediated Apoptosis Signaling
CXCR4 Signaling	Axonal Guidance Signaling
14-3-3-mediated Signaling	Glioblastoma Multiforme Signaling
Actin Nucleation by ARP-WASP Complex	3-phosphoinositide Degradation
Role of NFAT in Regulation of the Immune Response	Epithelial Adherens Junction Signaling
RhoGDI Signaling	Aldosterone Signaling in Epithelial Cells
VEGF Signaling	NGF Signaling
Cardiac Hypertrophy Signaling	3-phosphoinositide Biosynthesis
Protein Kinase A Signaling	G Protein Signaling Mediated by Tubby
Cholecystokinin/Gastrin-mediated Signaling	Role of Tissue Factor in Cancer
Breast Cancer Regulation by Stathmin1	PEDF Signaling
D-myo-inositol-5-phosphate Metabolism	Cdc42 Signaling
Gαq Signaling	Gα12/13 Signaling
Chemokine Signaling	P2Y Purigenic Receptor Signaling Pathway
JAK/Stat Signaling	FLT3 Signaling in Hematopoietic Progenitor Cells
Ephrin B Signaling	CREB Signaling in Neurons
Integrin Signaling	Huntington's Disease Signaling
CCR3 Signaling in Eosinophils	PI3K/AKT Signaling
p70S6K Signaling	Role of NFAT in Cardiac Hypertrophy
Actin Cytoskeleton Signaling	Phospholipase C Signaling
RhoA Signaling	Melanoma Signaling
Cell Cycle: G2/M DNA Damage Checkpoint Regulation	Regulation of the Epithelial-Mesenchymal Transition Pathway
HIPPO signaling	Prostate Cancer Signaling
D-myo-inositol (1,4,5,6)-Tetrakisphosphate Biosynthesis	Ovarian Cancer Signaling
D-myo-inositol (3,4,5,6)-tetrakisphosphate Biosynthesis	Insulin Receptor Signaling
G Beta Gamma Signaling	Asparagine Biosynthesis I
Signaling by Rho Family GTPases	Synaptic Long Term Depression
IL-8 Signaling	Ephrin A Signaling
Role of Macrophages, Fibroblasts and Endothelial Cells in Rheumatoid Arthritis	Chronic Myeloid Leukemia Signaling
Mouse Embryonic Stem Cell Pluripotency	

*P<0.01 for the pathways listed; pathways are listed from lowest p value to greatest p value. The BMSC lots with high bone formation scores were from Centers 2 and 6 and those with low bone formation scores were from Centers 4 and 5.

Supplementary Table S4. Number of aliquots from each BMSC lot tested in the *in vivo* transplant model

<table>
<tr><th rowspan="2">Center</th><th rowspan="2">Lot</th><th colspan="2">Transplants number</th></tr>
<tr><th>8 weeks</th><th>16 weeks</th></tr>
<tr><td rowspan="3">#1</td><td>A</td><td rowspan="3">3</td><td rowspan="3">3</td></tr>
<tr><td>B</td></tr>
<tr><td>C</td></tr>
<tr><td rowspan="3">#2</td><td>A</td><td rowspan="3">3</td><td rowspan="3">3</td></tr>
<tr><td>B</td></tr>
<tr><td>C</td></tr>
<tr><td rowspan="2">#3</td><td>A</td><td rowspan="2">No</td><td rowspan="2">No</td></tr>
<tr><td>B</td></tr>
<tr><td rowspan="3">#4</td><td>A</td><td rowspan="2">2</td><td rowspan="2">3</td></tr>
<tr><td>B</td></tr>
<tr><td>C</td><td>No</td><td>No</td></tr>
<tr><td rowspan="3">#5</td><td>A</td><td rowspan="2">No</td><td rowspan="2">1</td></tr>
<tr><td>B</td></tr>
<tr><td>C</td><td>3</td><td>3</td></tr>
<tr><td rowspan="4">#6</td><td>A</td><td rowspan="3">3</td><td rowspan="3">3</td></tr>
<tr><td>B</td></tr>
<tr><td>C</td></tr>
<tr><td>D</td><td>No</td><td>No</td></tr>
<tr><td rowspan="3">#7</td><td>A</td><td rowspan="3">No</td><td rowspan="3">No</td></tr>
<tr><td>B</td></tr>
<tr><td>C</td></tr>
<tr><td rowspan="3">#8</td><td>A</td><td rowspan="3">3</td><td rowspan="3">3</td></tr>
<tr><td>B</td></tr>
<tr><td>C</td></tr>
</table>

Three replicates per sample were performed when the number of cells provided by the centers was sufficient. For those samples with a lower number of cells, 16 weeks transplants were prioritized.

Supplementary Table S5. Marrow Adiposity and Hematopoiesis Score System

Adiposity Score	Hematopoiesis Score	Adipose tissue relative area (A)	Observations
0	4	A ≤10%	Few or no adipocytes present
1	3	10%< A ≤40%	Marrow cavities look mostly blue, with sparse adipocytes inside
2	2	40%< A ≤60%	Marrow cavities show approximately the same amount of hematopoietic and adipose tissue
3	1	60%< A ≤90%	Hematopoietic cells seem to occupy the spaces left free by the adipocytes
4	0	A ≥90%	It requires an effort to find hematopoietic cells in the cavities

Reference List

1. Ren,J. *et al.* Intra-subject variability in human bone marrow stromal cell (BMSC) replicative senescence: Molecular changes associated with BMSC senescence. *Stem Cell Res.* **11**, 1060-1073 (2013).

2. Bronckaers,A. *et al.* Mesenchymal stem/stromal cells as a pharmacological and therapeutic approach to accelerate angiogenesis. *Pharmacol. Ther.*(2014).

3. Boxall,S.A. & Jones,E. Markers for characterization of bone marrow multipotential stromal cells. *Stem Cells Int.* **2012**, 975871 (2012).

4. Jin,P. *et al.* Direct T cell-tumour interaction triggers TH1 phenotype activation through the modification of the mesenchymal stromal cells transcriptional programme. *Br. J. Cancer* **110**, 2955-2964 (2014).

5. Gibon,E. *et al.* Effect of a CCR1 receptor antagonist on systemic trafficking of MSCs and polyethylene particle-associated bone loss. *Biomaterials* **33**, 3632-3638 (2012).

6. Guang,L.G., Boskey,A.L., & Zhu,W. Age-related CXC chemokine receptor-4-deficiency impairs osteogenic differentiation potency of mouse bone marrow mesenchymal stromal stem cells. *Int. J. Biochem. Cell Biol.* **45**, 1813-1820 (2013).

7. Mailloux,A.W. *et al.* Fibrosis and subsequent cytopenias are associated with basic fibroblast growth factor-deficient pluripotent mesenchymal stromal cells in large granular lymphocyte leukemia. *J. Immunol.* **191**, 3578-3593 (2013).

8. Sioud,M., Mobergslien,A., Boudabous,A., & Floisand,Y. Mesenchymal stem cell-mediated T cell suppression occurs through secreted galectins. *Int. J. Oncol.* **38**, 385-390 (2011).

9. Sioud,M., Mobergslien,A., Boudabous,A., & Floisand,Y. Evidence for the involvement of galectin-3 in mesenchymal stem cell suppression of allogeneic T-cell proliferation. *Scand. J. Immunol.* **71**, 267-274 (2010).

10. Gieseke,F. *et al.* Proinflammatory stimuli induce galectin-9 in human mesenchymal stromal cells to suppress T-cell proliferation. *Eur. J. Immunol.* **43**, 2741-2749 (2013).

11. Plotnikov,E.Y. *et al.* Inflammatory pre-conditioning of mesenchymal multipotent stromal cells improves their immunomodulatory potency in acute pyelonephritis in rats. *Cytotherapy.* **15**, 679-689 (2013).

12. Chabannes,D. *et al.* A role for heme oxygenase-1 in the immunosuppressive effect of adult rat and human mesenchymal stem cells. *Blood* **110**, 3691-3694 (2007).

13. Oyanagi,J. *et al.* Inhibition of transforming growth factor-beta signaling potentiates tumor cell invasion into collagen matrix induced by fibroblast-derived hepatocyte growth factor. *Exp. Cell Res.*(2014).

14. Selmani,Z. *et al.* Human leukocyte antigen-G5 secretion by human mesenchymal stem cells is required to suppress T lymphocyte and natural killer function and to induce CD4+CD25highFOXP3+ regulatory T cells. *Stem Cells* **26**, 212-222 (2008).

15. Meisel,R. *et al.* Human bone marrow stromal cells inhibit allogeneic T-cell responses by indoleamine 2,3-dioxygenase-mediated tryptophan degradation. *Blood* **103**, 4619-4621 (2004).

16. Peng,Y. *et al.* Mesenchymal stromal cells infusions improve refractory chronic graft versus host disease through an increase of CD5+ regulatory B cells producing interleukin 10. *Leukemia*(2014).

17. Goolaerts,A. *et al.* Conditioned media from mesenchymal stromal cells restores sodium transport and preserves epithelial permeability in an in vitro model of acute alveolar injury. *Am. J. Physiol Lung Cell Mol. Physiol*(2014).

18. Nasef,A. *et al.* Leukemia inhibitory factor: Role in human mesenchymal stem cells mediated immunosuppression. *Cell Immunol.* **253**, 16-22 (2008).

19. Russell,K.C. *et al.* Cell-surface expression of neuron-glial antigen 2 (NG2) and melanoma cell adhesion molecule (CD146) in heterogeneous cultures of marrow-derived mesenchymal stem cells. *Tissue Eng Part A* **19**, 2253-2266 (2013).

20. Lee,K.S. *et al.* Effects of serial passage on the characteristics and chondrogenic differentiation of canine umbilical cord matrix derived mesenchymal stem cells. *Asian-Australas. J. Anim Sci.* **26**, 588-595 (2013).

21. Dong,W. *et al.* Phenotypic characterization of craniofacial bone marrow stromal cells: unique properties of enhanced osteogenesis, cell recruitment, autophagy, and apoptosis resistance. *Cell Tissue Res.*(2014).

22. De,B.C. *et al.* A biomarker-based mathematical model to predict bone-forming potency of human synovial and periosteal mesenchymal stem cells. *Arthritis Rheum.* **58**, 240-250 (2008).

23. Ball,S.G., Shuttleworth,A., & Kielty,C.M. Inhibition of platelet-derived growth factor receptor signaling regulates Oct4 and Nanog expression, cell shape, and mesenchymal stem cell potency. *Stem Cells* **30**, 548-560 (2012).

24. Solchaga,L.A. & Zale,E.A. Prostaglandin E2: a putative potency indicator of the immunosuppressive activity of human mesenchymal stem cells. *Am. J. Stem Cells* **1**, 138-145 (2012).

25. Menge,T. *et al.* Mesenchymal stem cells regulate blood-brain barrier integrity through TIMP3 release after traumatic brain injury. *Sci. Transl. Med.* **4**, 161ra150 (2012).

26. Lombardo,E. *et al.* Toll-like receptor-mediated signaling in human adipose-derived stem cells: implications for immunogenicity and immunosuppressive potential. *Tissue Eng Part A* **15**, 1579-1589 (2009).

27. DelaRosa,O., Dalemans,W., & Lombardo,E. Toll-like receptors as modulators of mesenchymal stem cells. *Front Immunol.* **3**, 182 (2012).

28. Lee,R.H. *et al.* Intravenous hMSCs improve myocardial infarction in mice because cells embolized in lung are activated to secrete the anti-inflammatory protein TSG-6. *Cell Stem Cell* **5**, 54-63 (2009).

29. Zisa,D., Shabbir,A., Suzuki,G., & Lee,T. Vascular endothelial growth factor (VEGF) as a key therapeutic trophic factor in bone marrow mesenchymal stem cell-mediated cardiac repair. *Biochem. Biophys. Res. Commun.* **390**, 834-838 (2009).

30. Chang,Y.S. *et al.* Critical role of VEGF secreted by mesenchymal stem cells in hyperoxic lung injury. *Am. J. Respir. Cell Mol. Biol.*(2014).

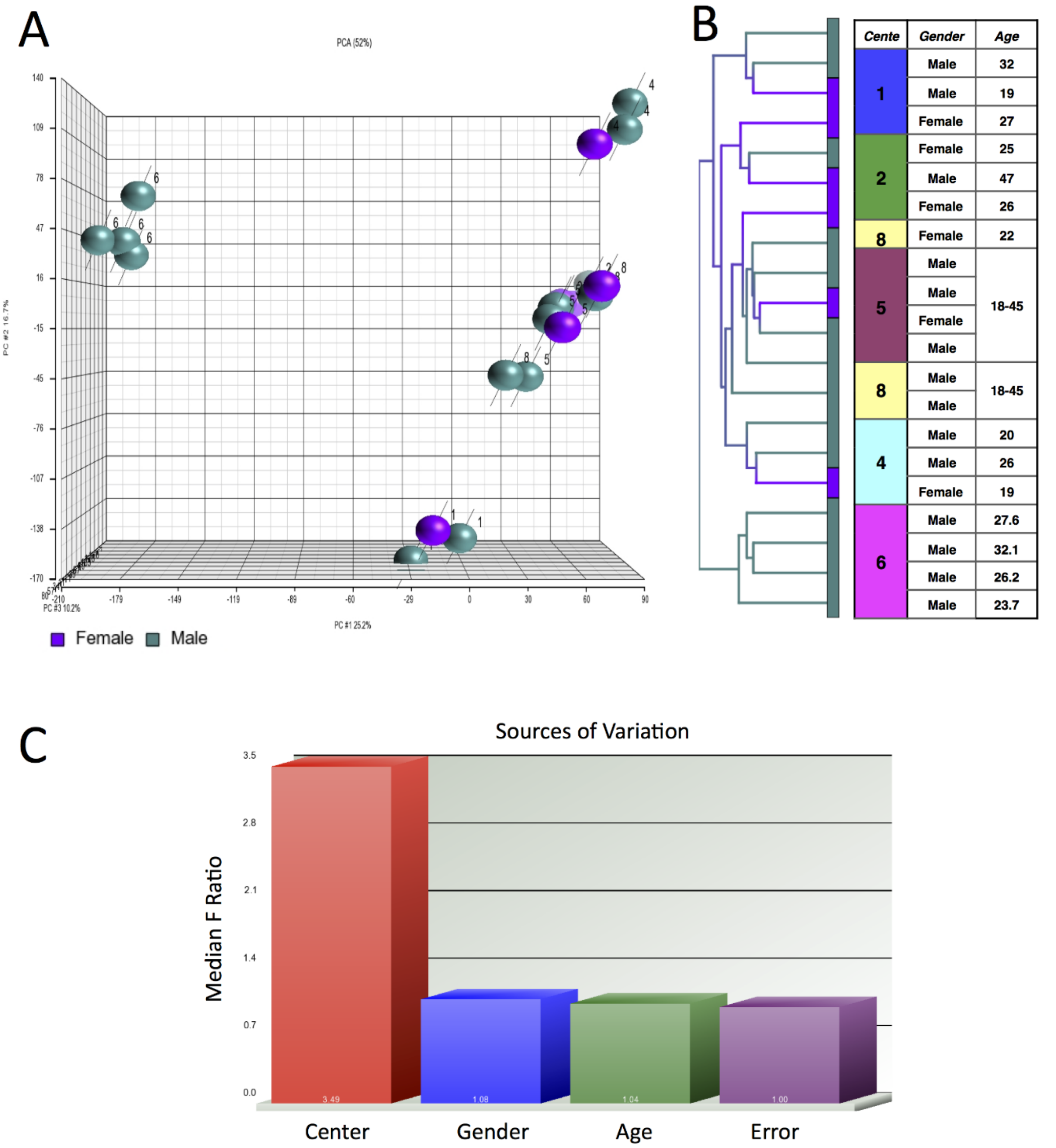

Supplementary Figure S1. Effects of bone marrow donor gender and age on BMSC characteristics. Specific information concerning bone marrow donor gender was available for 20 BMSC lots and age for 14 of the 20. Results of analysis of gene expression data from the 20 lots by PCA are shown in panel A and by unsupervised hierarchical clustering analysis in panel B. Results of analysis of the gene expression data from the 14 lots by 3-way ANOVA are shown in panel C.

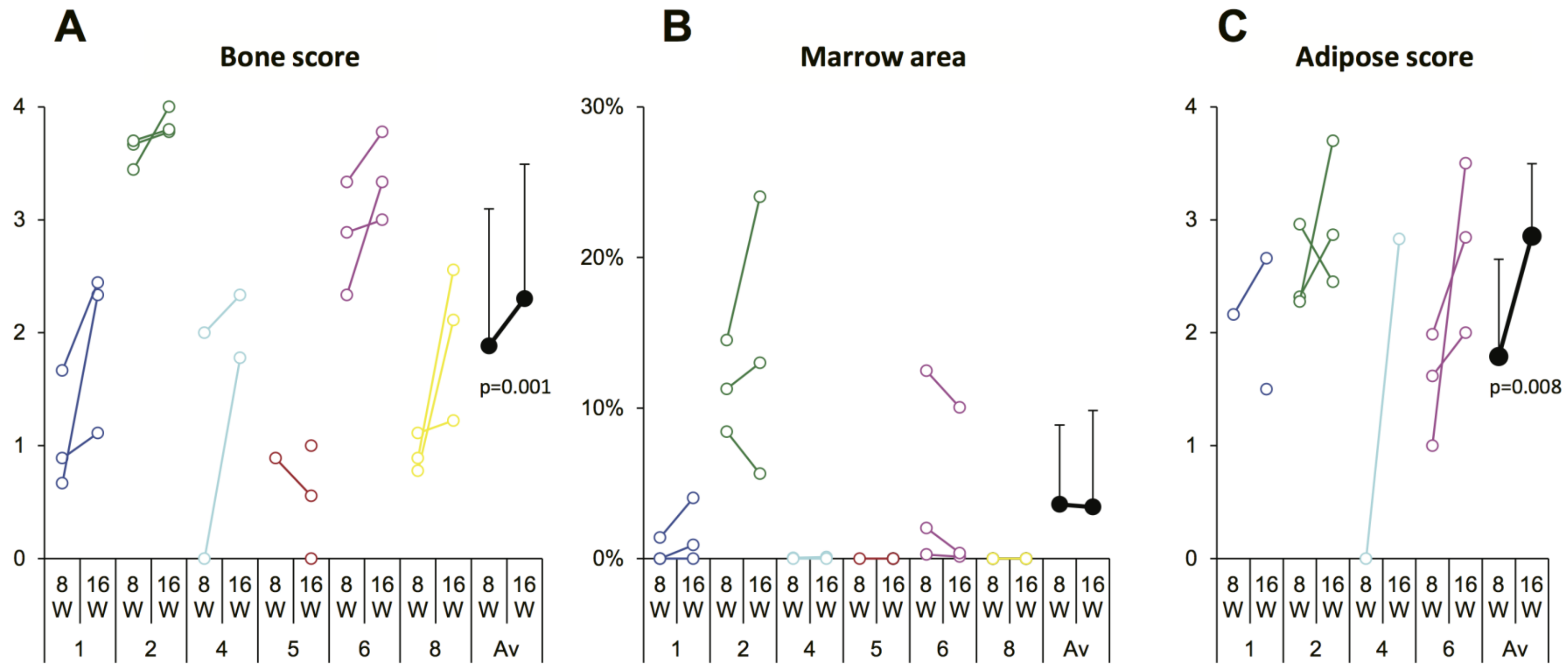

Supplementary Figure S2. Change in BMSC transplant results between 8 and 16 weeks. The bone formation score (panel A), marrow area coverage (panel B) and adipose tissue coverage (panel C) at week 8 and 16 for each BMSC lot is shown. The manufacturing center number and week post-transplant are shown at the bottom of the figure.

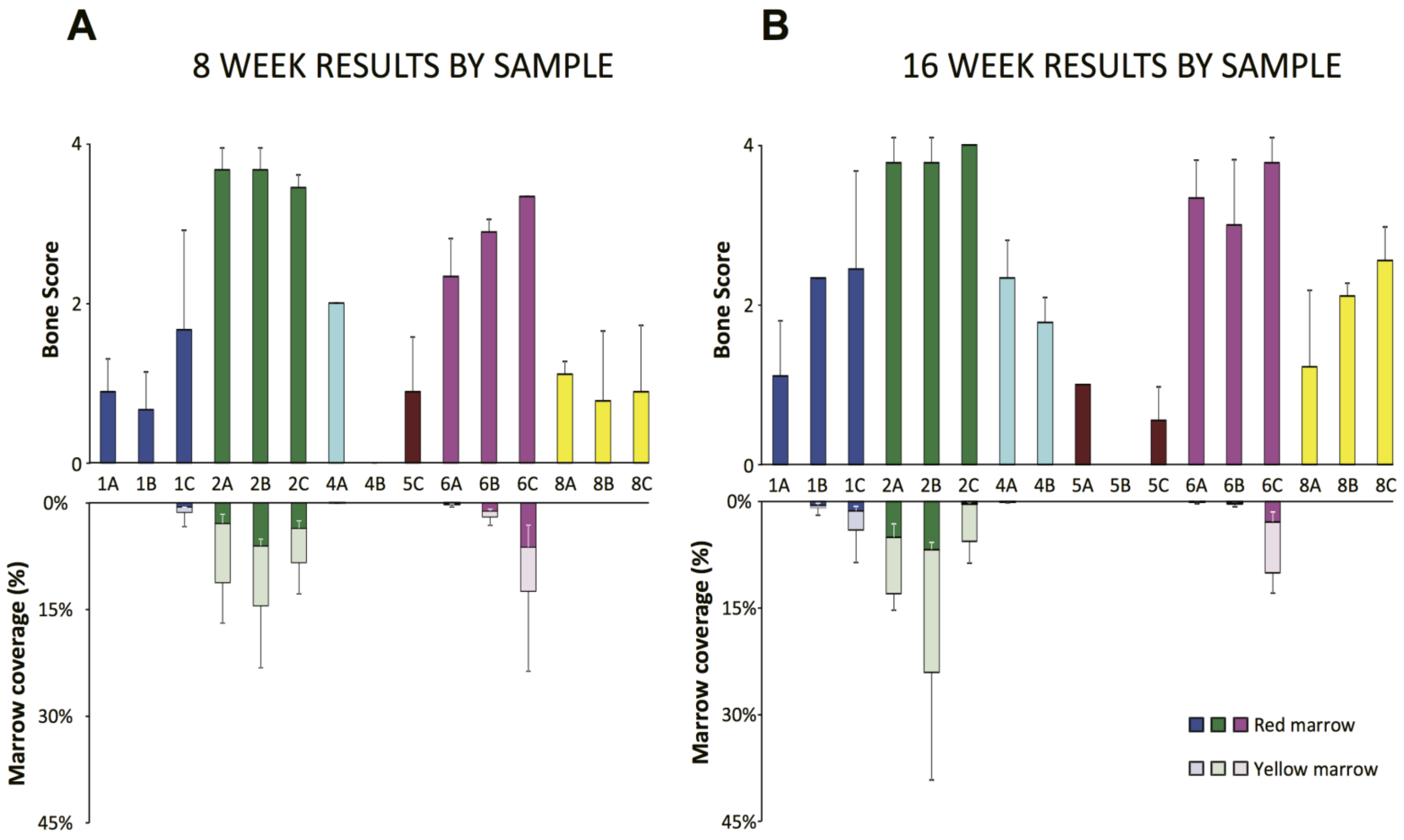

Supplementary Figure S3. Bone formation, support of hematopoiesis and adipose tissue formation for each BMSC lot tested. Each BMSC lot was evaluated after 8 and 16 weeks for bone formation and area of marrow coverage. The bone formation scores are shown in the upper portion of each panel and the area of marrow coverage, hematopoietic progenitor cell coverage score (red marrow) and adipose tissue coverage score (yellow marrow) are shown in the lower portion of each panel. The results of analysis 8 weeks after transplantation are shown in panel A and 16 weeks after transplantation in panel B. For BMSCs from Centers #1, #2, #6 and #8, three BMSC lots were tested at both time points. For Center #4 two BMSC lots were tested at 8 weeks and 2 at 16 weeks, and for Center #5, one BMSC lot was tested at 8 weeks, and at 16 weeks three lots were tested. The values shown represent the mean ± 1 SD.

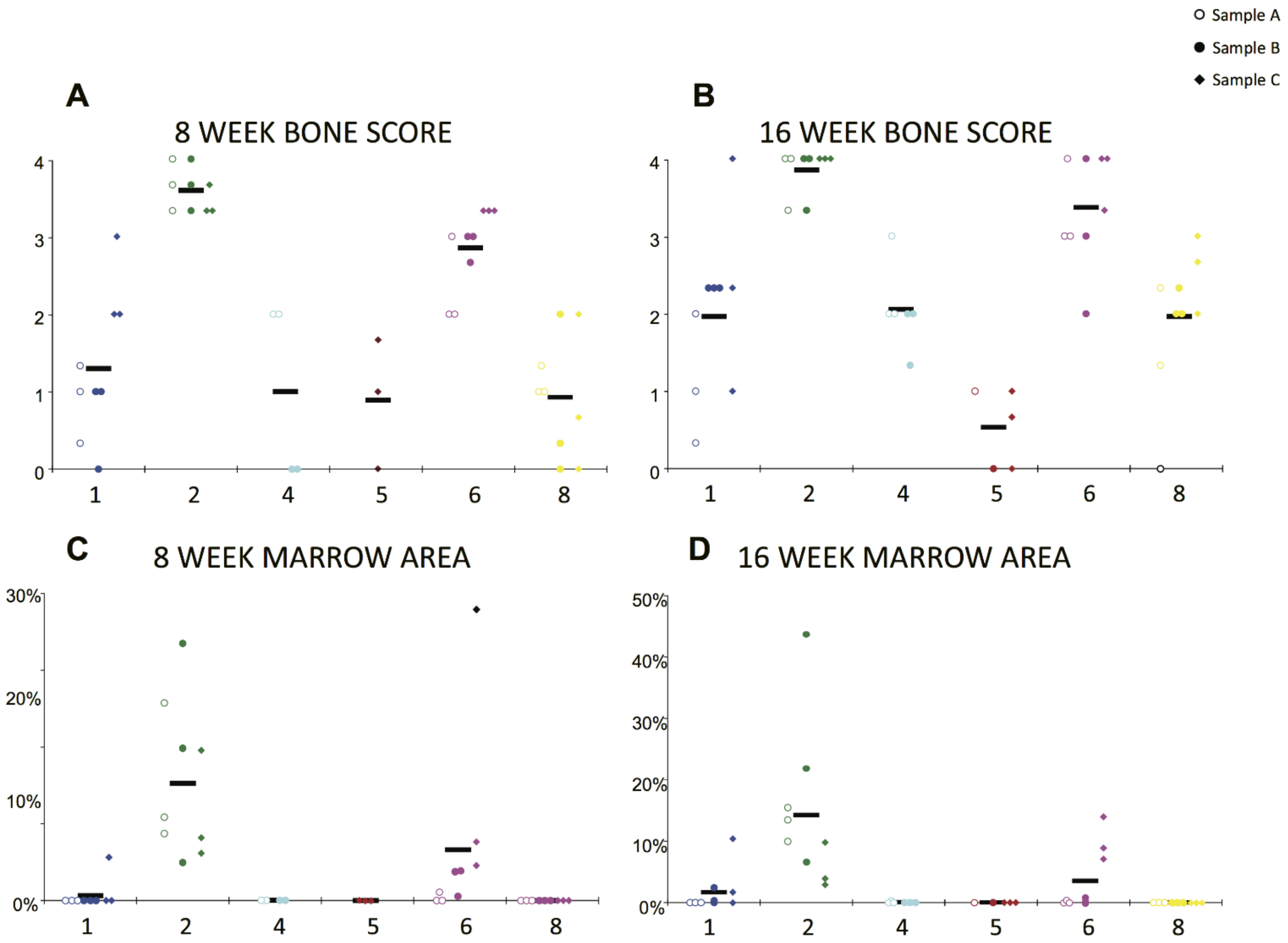

Supplementary Figure S4. Replicate bone formation, support of hematopoiesis and adipose tissue formation for each BMSC lot tested. Each BMSC lot was tested in 1 to 3 separate transplant assays for bone formation and marrow area. Each sample was evaluated after 8 and 16 weeks for bone formation and area of marrow coverage. The bone formation scores are shown in the upper portion of each panel and the area of marrow coverage, hematopoietic progenitor cell coverage score (red marrow) and adipose tissue coverage score (yellow marrow) are shown in the lower portion of each panel. The results of analysis 8 weeks after transplantation are shown in panel A and 16 weeks after transplantation in panel B. The values shown represent the mean ± 1 SD.

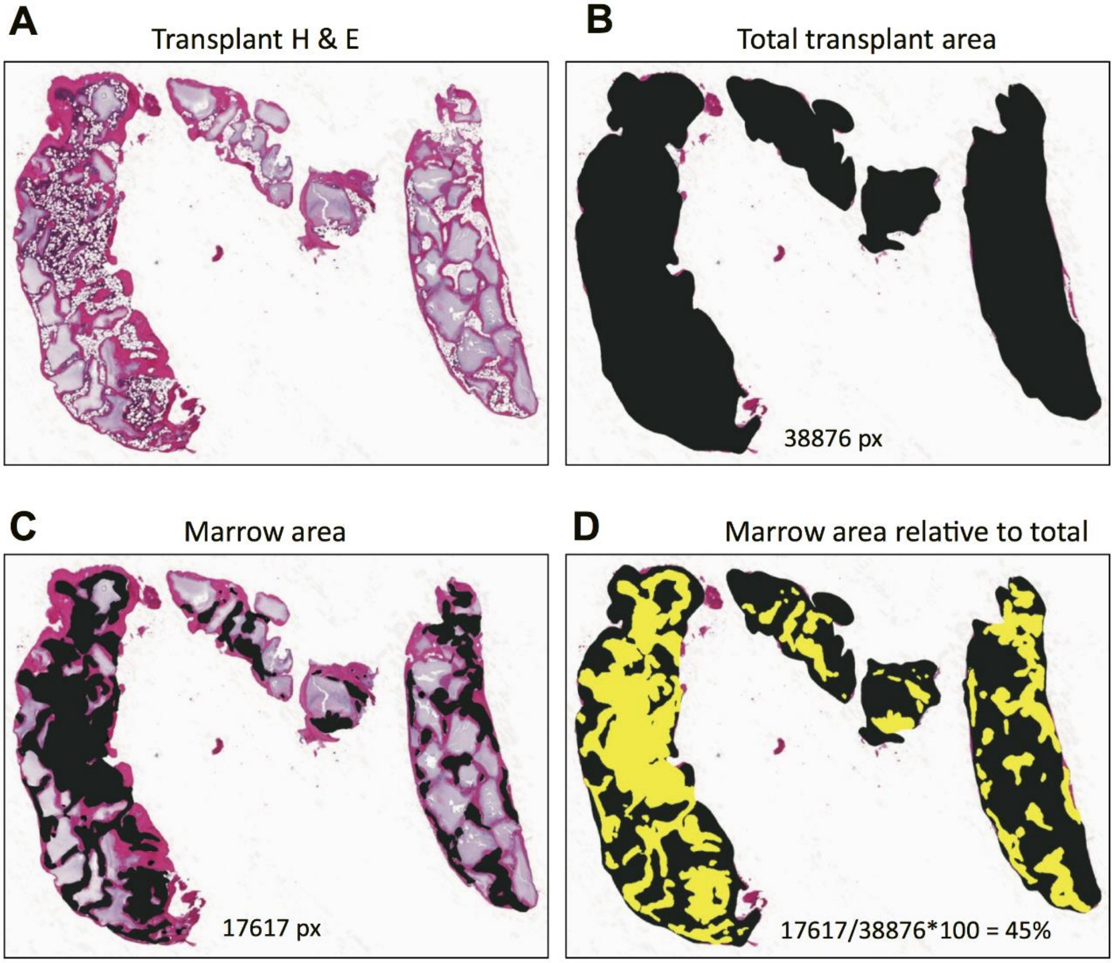

Supplementary Figure S5. Relative content of marrow produced by transplanted BMSCs. The relative content of marrow in each transplant was quantified in low magnification images by manually contouring the area covered by marrow and the total surface of the transplant using Adobe Photoshop CS6. Hematoxylin and Eosin staining of transplanted BMSCs is show in panel A. The total area of the transplant is show in panel B. The area covered by marrow is shown in panel C and the area of marrow coverage relative to the total area of the transplant is shown in panel D.

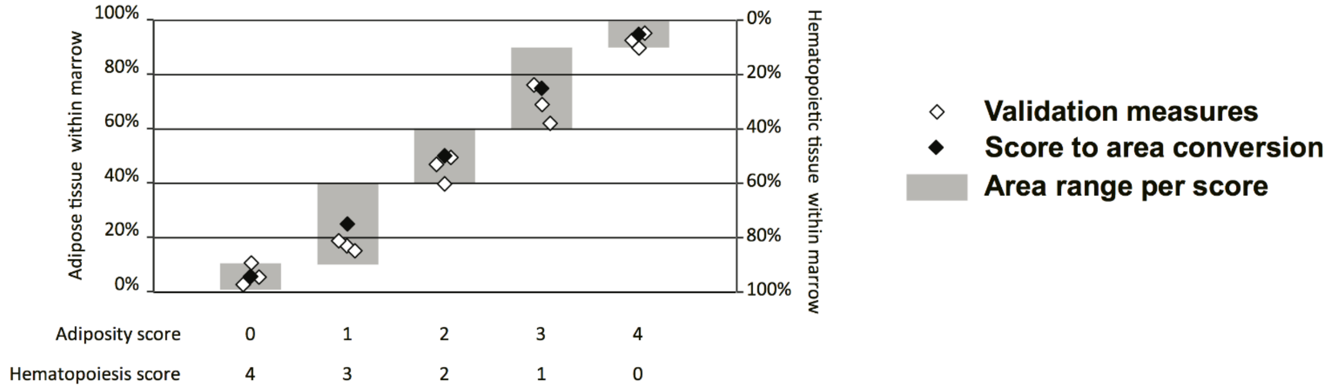

Supplementary Figure S6. Validation of the Marrow Adiposity, and Hematopoiesis Scores for determining the area of marrow coverage by adipose tissue. For each of 15 BMSC transplant micrographs, the area of coverage by adipose tissue was determined by using Adobe Photoshop and the adipocyte score described in Supplementary table 2. A comparison of the results of analysis of each of the 15 samples using the 2 methods is shown.
